# Supplementary figures and images for: From Many, One: Genetic Control of Prolificacy during Maize Domestication
Source: PLoS Genet. 2013 Jun 27;9(6):e1003604. doi: 10.1371/journal.pgen.1003604 (PMC3694832; doi:10.1371/journal.pgen.1003604)

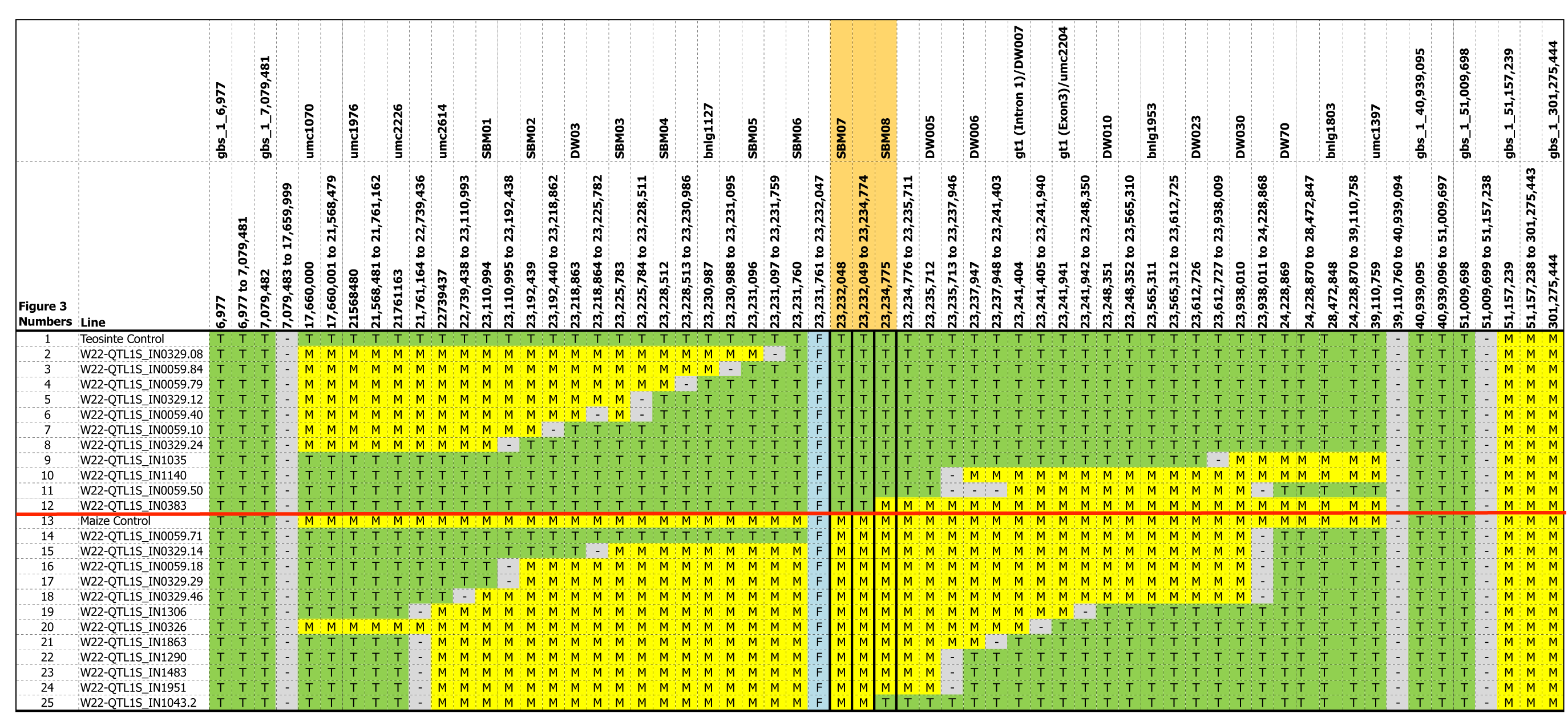

Supplement: Figure S1 — Breakpoints of introgressed teosinte chromosomal segments used in the substitution mapping of prol1.1. Positions shown at the top of each column are based on the B73 Maize reference genome AGP_v2. Details for the markers listed in the top row can be found in Table S1. Rows represent the 23 Recombinant Chromosome Lines plus the maize and teosinte control lines. The genotypes for markers and intervals of each line are shown: “M” = maize, “T” = teosinte, “−” = undetermined, and “F” = fixed such that the maize and teosinte sequences are identical in the interval. The causative interval is highlighted in orange. (TIF) [file pgen.1003604.s001.tif]

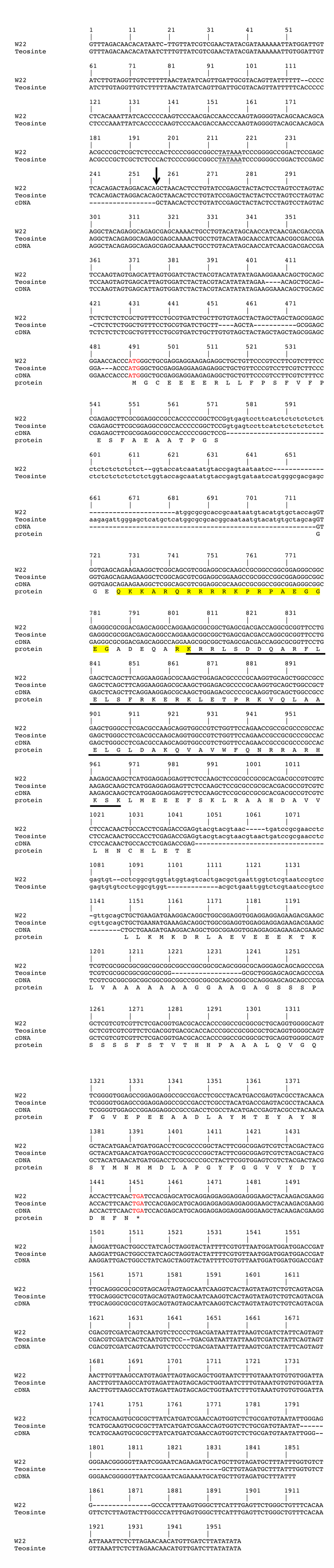

Supplement: Figure S2 — Gene model for grassy tillers1 (gt1) inferred from our maize (W22) and teosinte genomic sequence and full length ESTs obtained from Genbank (EB673843, DV519626). The following inferred features are marked: TATA box (underlined), transcription start (bold arrow), translation start (red text), introns (lower case), nuclear localization signal (yellow highlight), homehbox (bold horizontal line), stop codon (red text). The nuclear localization signal was predicted using the software NLStradamus (http://www.moseslab.csb.utoronto.ca/NLStradamus/). (TIF) [file pgen.1003604.s002.tif]

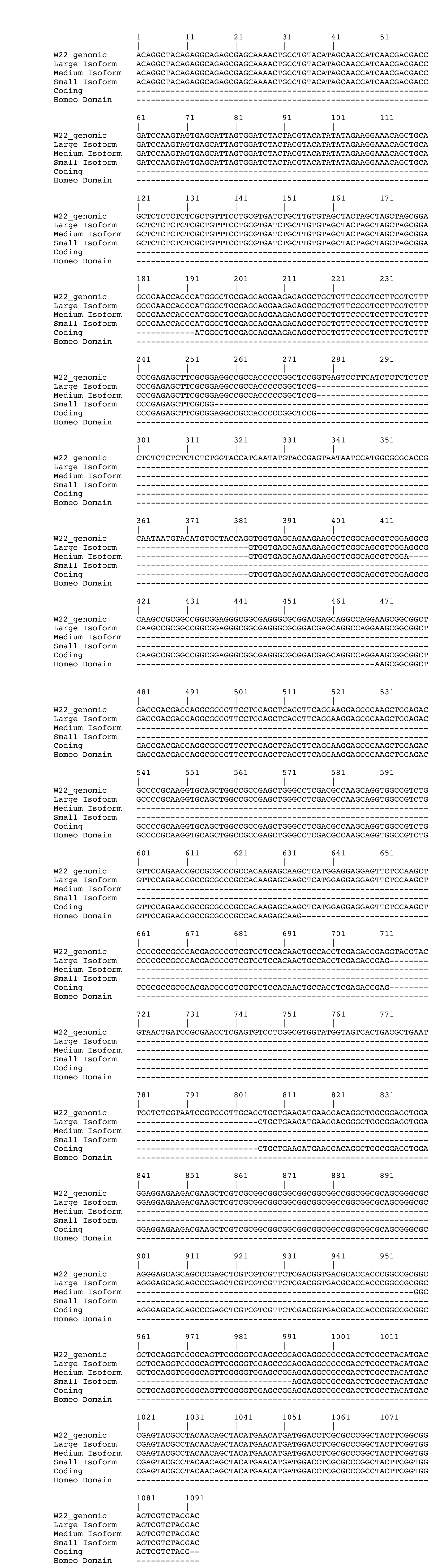

Supplement: Figure S3 — Nucleotide sequences for the large, medium and small RT-PCR products aligned with W22 maize genomic sequence, gt1 coding sequence, and sequence for the homeodomain. (TIF) [file pgen.1003604.s003.tif]

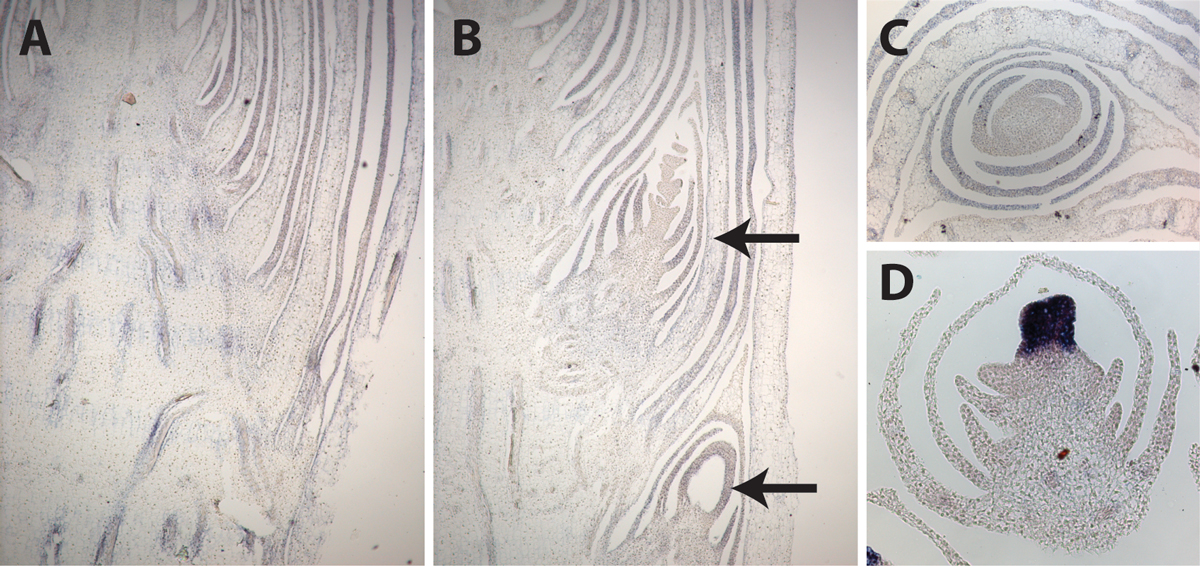

Supplement: Figure S4 — (A) Longitudinal section of a primary lateral branch from the M:M genotype, hybridized with antisense gt1 RNA probe. gt1 is expressed at low levels in the nodes of the primary branch. No secondary branch buds have initiated. The M:T genotype also showed nodal expression and a lack of secondary bud initiation. (B) Primary lateral branch from the T:T genotype, hybridized with gt1 probe. No nodal gt1 expression is observed. Two prominent secondary branch buds have initiated and are actively growing (arrows). (C) Transverse section of a secondary lateral branch, shows low levels of gt1 expressed in the bud leaves. Weak gt1 expression in secondary branches suggests that these buds are not dormant. (D) Control hybridization of gt1 to a male floret shows strong gt1 hybridization to cells in the arrested carpel primordium. This strong gt1 expression is evident within 3–4 hours after the initiation of the color reaction, while the weaker gt1 expression in secondary lateral branch leaves or in the node requires 15–20 hours for detection (see methods). (TIF) [file pgen.1003604.s004.tif]

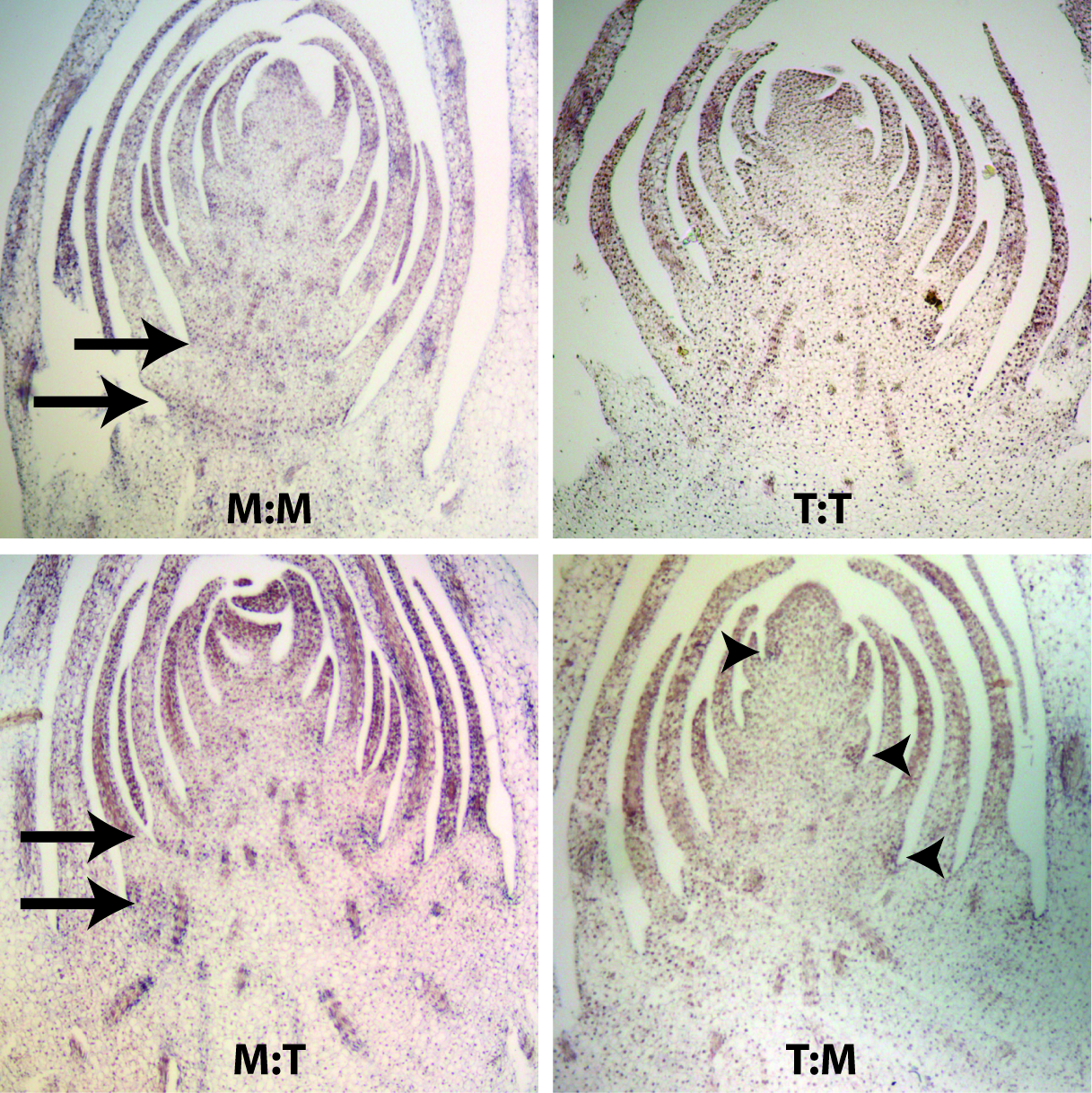

Supplement: Figure S5 — gt1 expression in young primary ear branches before the floral transition. Consistent with the expression observed in older ear branches (main text Fig. 5), gt1 is present in the nodes (arrows) of lines containing the maize control region (M:M and M:T), but absent from those lines that have the teosinte control region (T:T and T:M). In addition, weak gt1 expression was observed in axillary buds (arrowheads). (TIF) [file pgen.1003604.s005.tif]

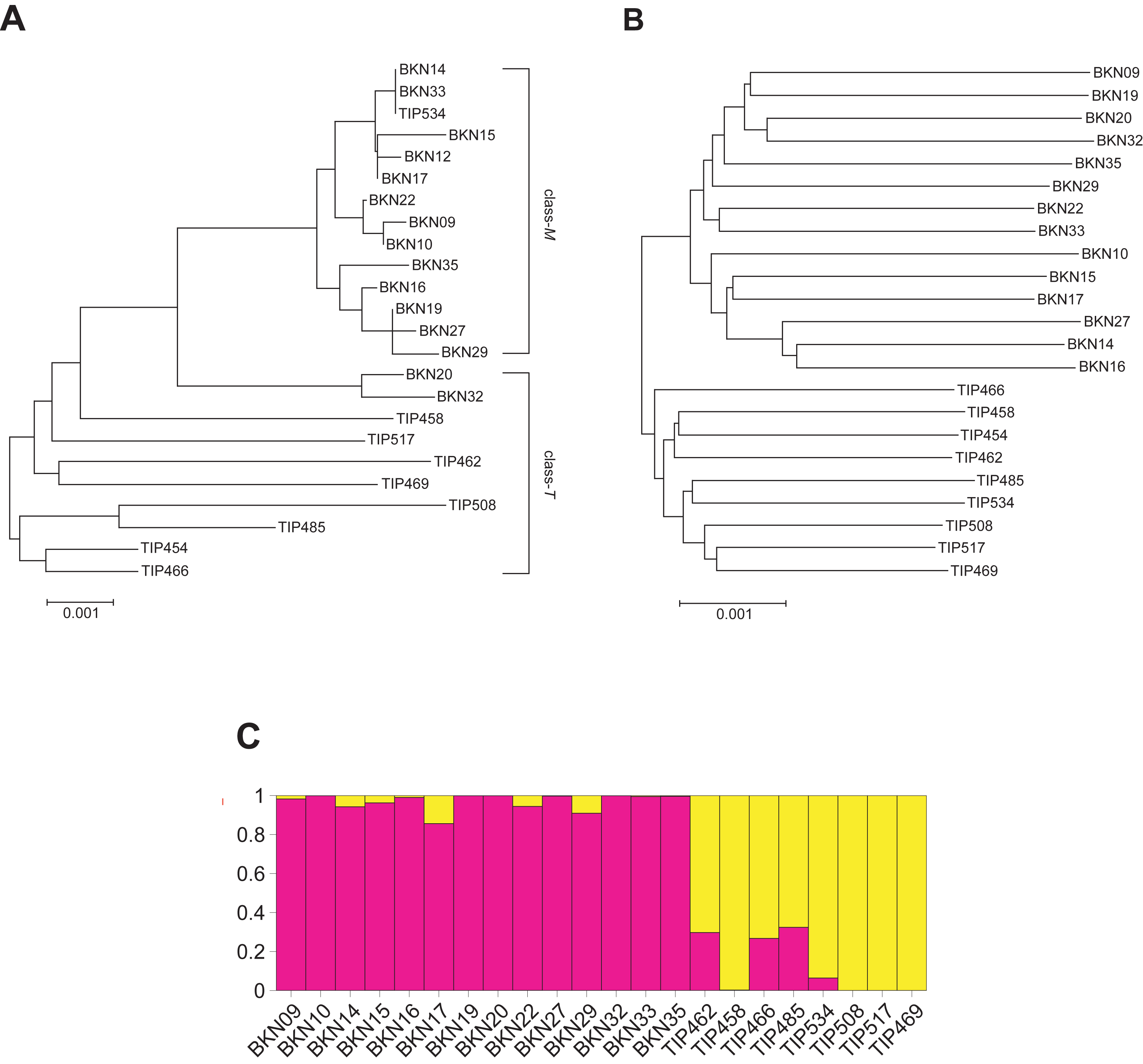

Supplement: Figure S6 — (A) Neighbor-joining tree in the QTL region. (B) Neighbor-joining tree inferred from the SNPs in chromosome 1. (C) The result of STRUCTURE analysis from the SNPs in chromosome 1. (TIF) [file pgen.1003604.s006.tif]

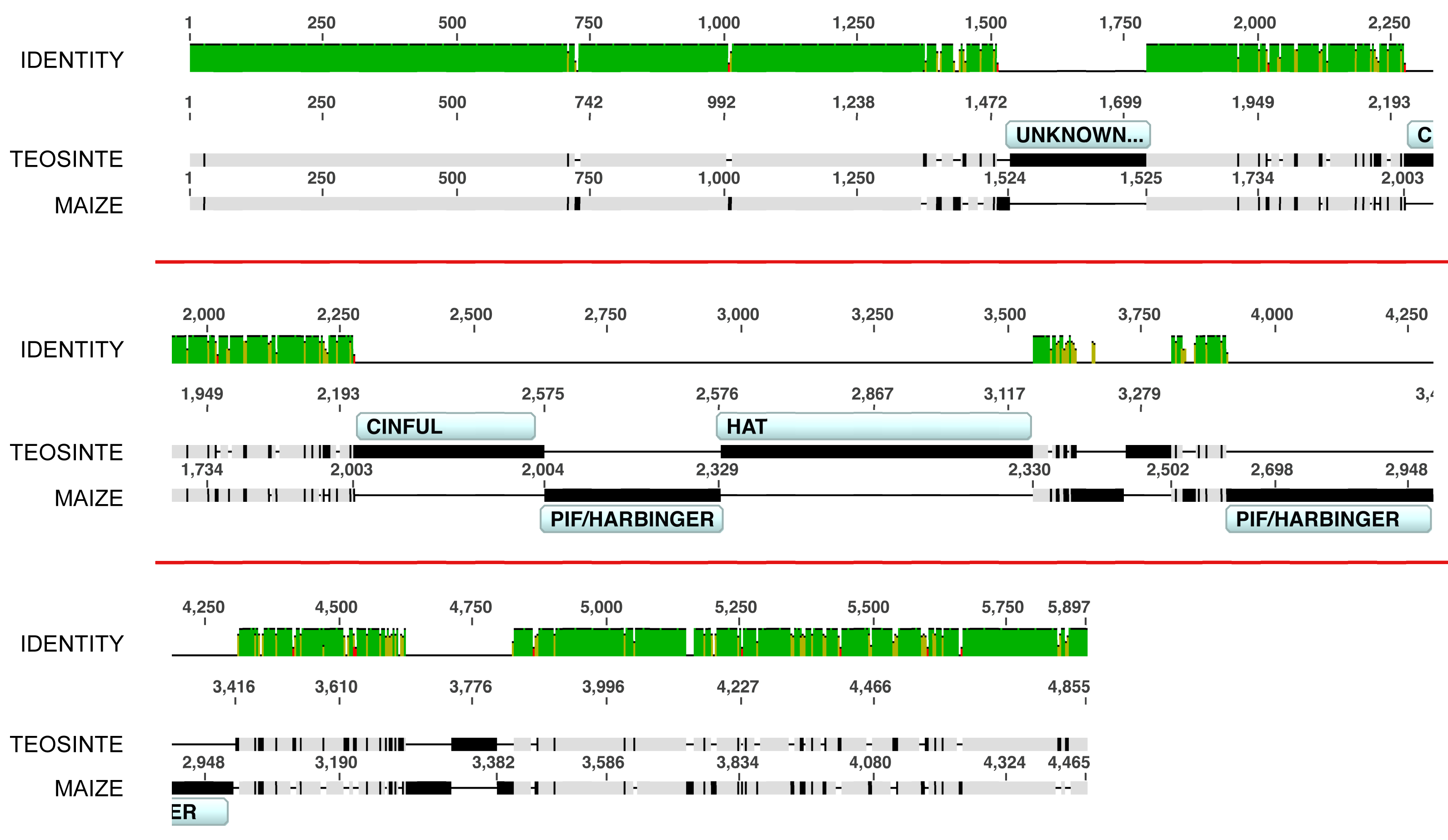

Supplement: Figure S7 — Alignment of the two parental lines showing annotation of large insertion/deletion (indel) polymorphisms. Large indels were annotated by BLAST against the maize transposable element database (maizetedb.org). The large indel annotated as “Unknown TE” showed small sections of homology to several transposable elements but no significant BLAST hits in the maize transposable element database or any sequence in Genbank. (TIF) [file pgen.1003604.s007.tif]

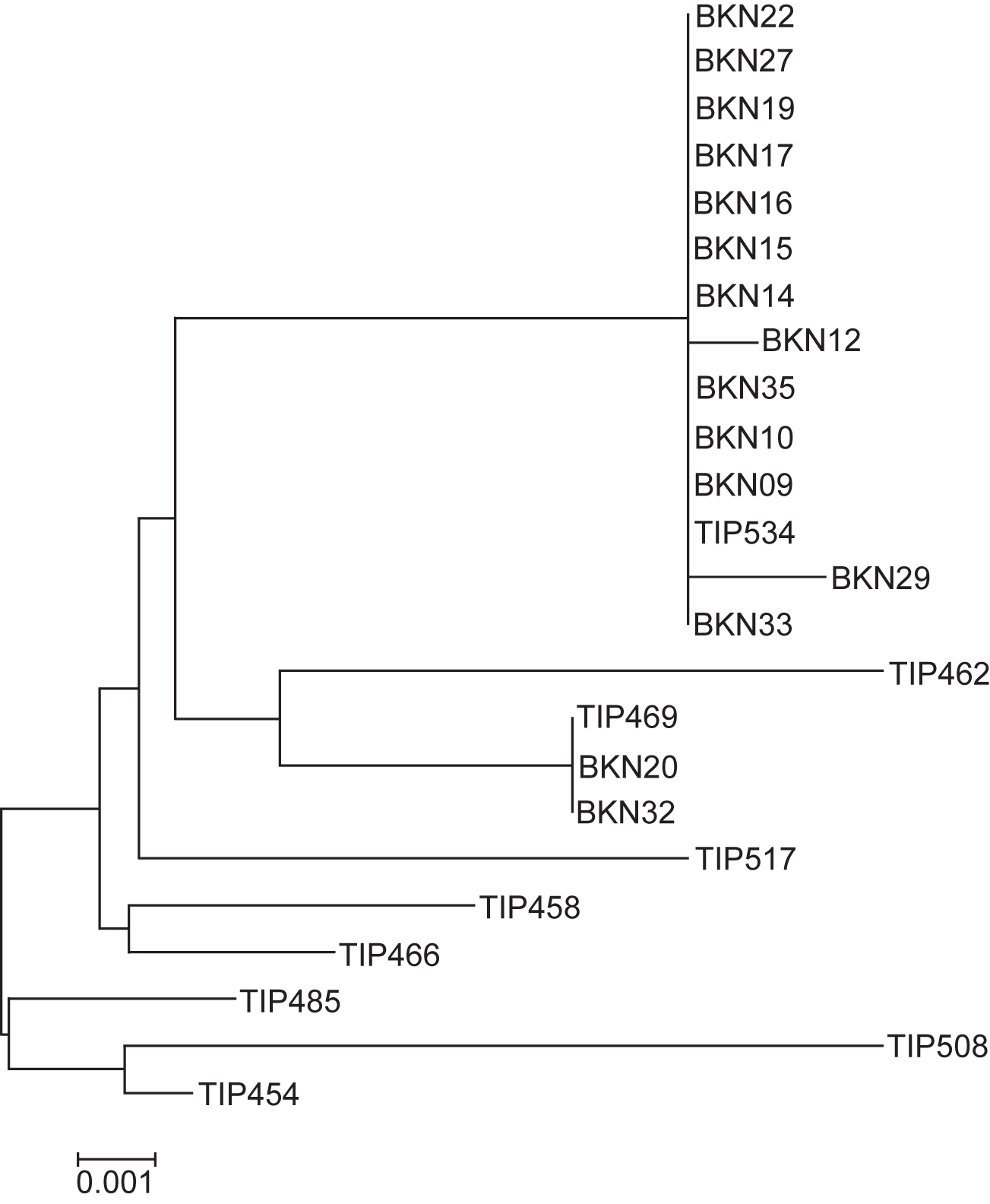

Supplement: Figure S8 — Neighbor-joining tree for the region-MLD. (TIF) [file pgen.1003604.s008.tif]

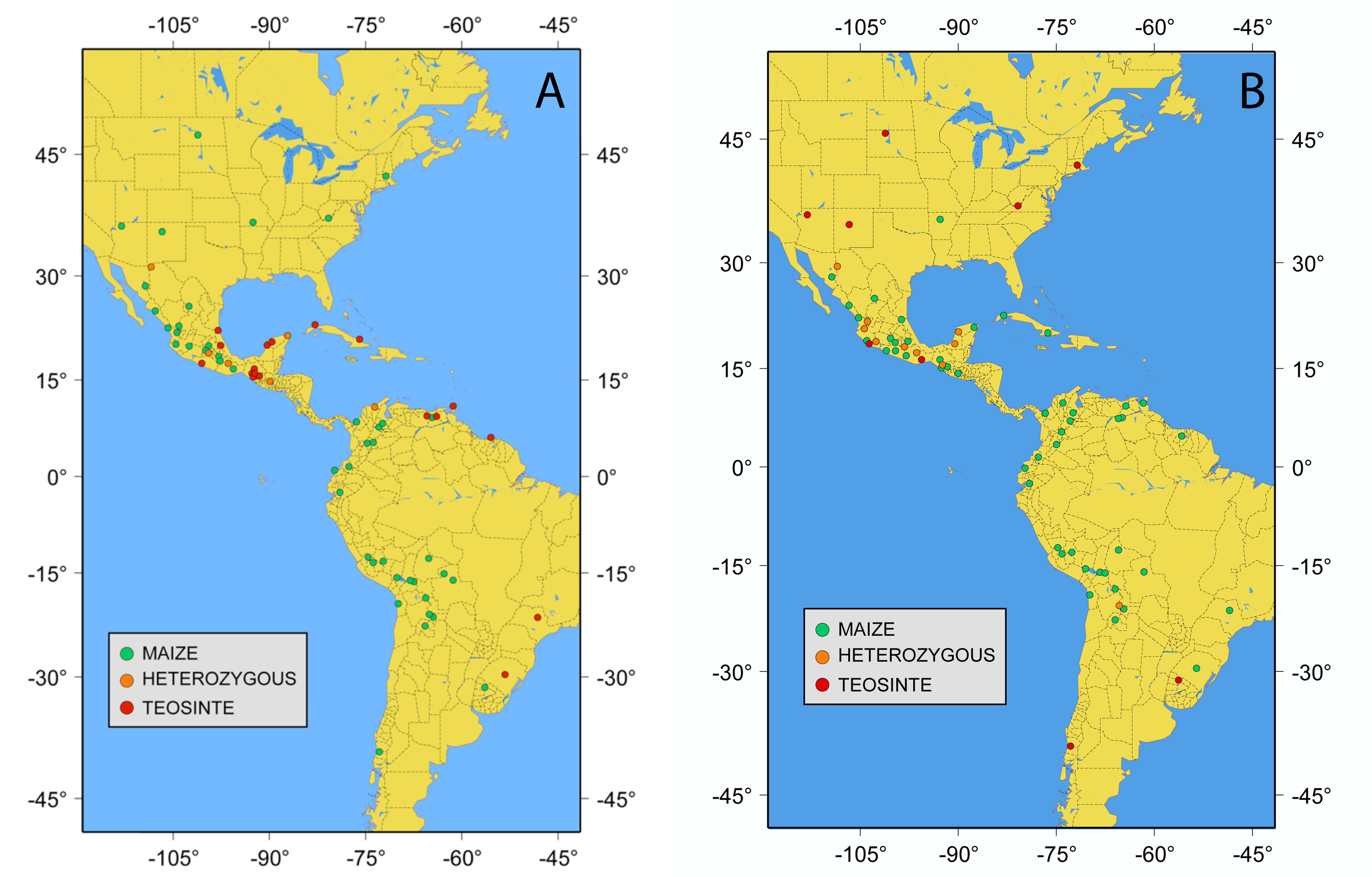

Supplement: Figure S9 — Distribution of the class-M and class-T haplotypes in maize landraces. (A) prol1.1. (B) gt1 3′ UTR. (TIF) [file pgen.1003604.s009.tif]
