# Supplementary material for: From Many, One: Genetic Control of Prolificacy during Maize Domestication
Source: PLoS Genet. 2013 Jun 27;9(6):e1003604. doi: 10.1371/journal.pgen.1003604 (PMC3694832; doi:10.1371/journal.pgen.1003604)
Supplement: Table S1 — Markers used to fine-map prol1.1. (DOCX) [file pgen.1003604.s010.docx]

| **Marker** | **Type**^a^ | **AGP_v2**  **Position** | **Polymorphism**  **(Maize/Teosinte)** | **Forward**  **Primer** | **Reverse**  **Primers** |
| --- | --- | --- | --- | --- | --- |
| gbs_1_6,977 | gbs | 6,977 |  |  |  |
| gbs_1_7,079,481 | gbs | 7,079,481 |  |  |  |
| umc1070 | SSR | 17,660,000^b^ | 180/199 bp | GGTCTCTCTATCGTCCGGTGAGTA | CCGGAGATGGGAAAGAAGATAAC |
| umc1976 | SSR | 21,568,480 | 79/89 bp | TGCCGAGGCTTCTAGTAGACCAA | CGCTATATCTATCCCGCAGCAAC |
| umc2226 | SSR | 21,761,163 | 81/84 bp | AGCTTCACGCTCTTCTAGACCAAA | TGCTGTGCAGTTCTTGCTTCTTAC |
| umc2614 | SSR | 22,739,437 | 162/163 bp | GTGTAGCTGATGATTCGAGCAAGA | ATACAACCATAGTATGCAACCGCC |
| SBM01 | SBM | 23,110,994 |  |  |  |
| SBM02 | SBM | 23,192,439 |  |  |  |
| DW03 | Indel | 23,218,863 | 10 bp Indel | TCGATAGCSAAGAGCAGGAT | CAAACTCGTCTGGAGATGGGGC |
| SBM03 | SBM | 23,225,783 |  |  |  |
| SBM04 | SBM | 23,228,512 |  |  |  |
| bnlg1127 | SSR | 23,230,987 | 117/109 bp | GCATGAGATGAGAGCAAAGC | TTCAAGGTGCAGTCTCATCG |
| SBM05 | SBM | 23,231,096 |  |  |  |
| SBM06 | SBM | 23,231,760 |  |  |  |
| SBM07 | SBM | 23,232,048 |  |  |  |
| SBM08 | SBM | 23,234,775 |  |  |  |
| DW05 | Indel | 23,235,712 | 3 bp Indel | CGCGCGCAAGGAAAAGTACACG | CCCTTCACGTGCTCGATCGTTC |
| DW06 | Indel | 23,237,947 | 5 bp Indel | AGCCAAGTGAGTTCAACCGCCA | TGCACAGGACGGTGGTGGGA |
| *grassy tillers1* intron1 (DW07) | Indel | 23,241,404 | 131/169 bp | CTCCGGTGAGTCCTTCATCT | AGCCTTCTTCTGCTCACCAC |
| *grassy tillers1* exon3  (umc2204) | SSR | 23,241,997 | 154/136 bp | AGCTGCTGAAGATGAAGGACAGG | TCACCGTCGAGAACGACGAC |
| DW10 | Indel | 23,248,351 | 4 bp Indel | TCAGGCATCTGACTTTTACCGACCA | GCAGCACGGCGTCTCCGAAA |
| bnlg1953 | SSR | 23,565,311 | 149/148 bp | CCTCGGAGCTCGATTTACAC | AACATTTAACCGCCGTCATC |
| DW23 | Indel | 23,614,011 | 3 bp Indel | GCGAGTCCACACCTTATCTACCGT | TGCTCGGTCAAGCACAGTCG |
| DW30 | Indel | 23,937,149 | 1 bp Indel | ACGCCCCACCCAATAGGTGAA | ACCAGGTGCCTGTGGCAGTC |
| DW70 | SSR | 24,228,869 | 165/168 bp | GACGTTGCCGGGGCTCACAC | GCTCCCACACTCGCCTCCCT |
| bnlg1803 | SSR | 28,472,848 | 202/210 bp | TGTTGTCTATTGGCAACCGA | GTATGCGTCGCTAGTCGTGA |
| umc1397 | SSR | 39,110,759 | 157/146 bp | GTTACACTTGCAGACAAACAACCG | GTCATGTGATCCGGGAGTAAATCT |
| gbs_1_40,939,095 | gbs | 40,939,095 |  |  |  |
| gbs_1_51,009,698 | gbs | 51,009,698 |  |  |  |
| gbs_1_51,157,239 | gbs | 51,157,239 |  |  |  |
| gbs_1_301,275,444 | gbs | 301,275,444 |  |  |  |

^a^gbs prefix (genotype by sequence) refers to marker from Next Generation sequencing of the 866 BC_2_S_3_ lines with further details posted at <http://www.panzea.org/lit/data_sets.html>. SBM prefix (sequence-based marker) is for marker genotypes determined by Sanger sequencing and applied only to the 23 recombinant chromosome progeny lines.

^b^Note: Precise AGP coordinates for umc1070 could not be identified in the B73 reference genome. Position was estimated based on the locations of flanking markers TIDP3567 and TIDP2777 on the IBM2 2008 Neighbors 1 map.
