# Supplementary material for: From Many, One: Genetic Control of Prolificacy during Maize Domestication
Source: PLoS Genet. 2013 Jun 27;9(6):e1003604. doi: 10.1371/journal.pgen.1003604 (PMC3694832; doi:10.1371/journal.pgen.1003604)
Supplement: Table S2 — Phenotypic effects on traits related to plant and ear architecture that are associated with prol1.1. (DOCX) [file pgen.1003604.s011.docx]

|  | **Genotype** | | |  |  |  |
| --- | --- | --- | --- | --- | --- | --- |
| **Trait** | **M/M** | **M/T** | **T/T** | **R^2^** | **N** | **p-value** |
| Prolificacy | 2.02 | 5.67 | 9.97 | 79.9 | 188 | 0.0001 |
| Number of ear branches | 3.56 | 3.49 | 3.62 | -0.6 | 185 | 0.6704 |
| Tillering | 1.61 | 1.79 | 1.68 | -0.2 | 181 | 0.4454 |
| Spikelet Number (top ear) | 418.1 | 422.6 | 420.6 | -0.9 | 174 | 0.8624 |
| 100 Kernel Wt (g) | 21.6 | 20.8 | 18.7 | 19.9 | 182 | 0.0001 |
