# Supplementary material for: From Many, One: Genetic Control of Prolificacy during Maize Domestication
Source: PLoS Genet. 2013 Jun 27;9(6):e1003604. doi: 10.1371/journal.pgen.1003604 (PMC3694832; doi:10.1371/journal.pgen.1003604)
Supplement: Table S3 — Number of visible initiated second ears observed on immature primary branches for the four possible combinations of maize (M) and teosinte (T) alleles at 5′ gt1 promoter and open reading frame (ORF). (DOCX) [file pgen.1003604.s012.docx]

| Genotype  (promoter:ORF) | Average Number of Secondary Buds per Primary Branch | Standard Deviation | Standard Error | n |
| --- | --- | --- | --- | --- |
| M:M | 0 | 0.000 | 0.000 | 6 |
| M:T | 0.25 | 0.500 | 0.250 | 4 |
| T:T | 2.5 | 0.837 | 0.342 | 6 |
| T:M | 3.8 | 1.10 | 0.490 | 5 |
