# Supplementary material for: From Many, One: Genetic Control of Prolificacy during Maize Domestication
Source: PLoS Genet. 2013 Jun 27;9(6):e1003604. doi: 10.1371/journal.pgen.1003604 (PMC3694832; doi:10.1371/journal.pgen.1003604)
Supplement: Table S4 — Samples used for DNA sequence analysis of diversity in the control region. (DOCX) [file pgen.1003604.s013.docx]

| **DNA Prep** | **Line/Accession** | **Type** | **Landrace/Species** | **Country** |
| --- | --- | --- | --- | --- |
| BKN09 | MR24 | Landrace Inbred Line | Chullpi | Peru |
| BKN10 | MR25 | Landrace Inbred Line | Pororo | Bolivia |
| BKN12 | MR01 | Landrace Inbred Line | Araguito | Venezuela |
| BKN14 | MR02 | Landrace Inbred Line | Assiniboine | USA |
| BKN15 | MR12 | Landrace Inbred Line | Havasupai | USA |
| BKN16 | MR14 | Landrace Inbred Line | Longfellow Flint | Canada |
| BKN17 | MR19 | Landrace Inbred Line | Santa Domingo | USA |
| BKN19 | MR05 | Landrace Inbred Line | Cateto | Bolivia |
| BKN20 | MR11 | Landrace Inbred Line | Cuban Flint | Cuba |
| BKN22 | MR18 | Landrace Inbred Line | Reventador | Mexico |
| BKN27 | MR10 | Landrace Inbred Line | Cristalino Norteno | Chile |
| BKN29 | MR03 | Landrace Inbred Line | Bolita | Mexico |
| BKN32 | MR09 | Landrace Inbred Line | Cravo Riogranense | Brazil |
| BKN33 | MR06 | Landrace Inbred Line | Chapalote | Mexico |
| BKN35 | MR21 | Landrace Inbred Line | Tabloncillo | Mexico |
| TIP454 | TIL04 | Inbred Line from Wild Species | Z. mays. ssp. parviglumis | Mexico |
| TIP458 | TIL05 | Inbred Line | Z. mays. ssp. parviglumis | Mexico |
| TIP462 | TIL02 | Inbred Line | Z. mays. ssp. parviglumis | Mexico |
| TIP466 | TIL07 | Inbred Line | Z. mays. ssp. parviglumis | Mexico |
| TIP469 | TIL16 | Inbred Line | Z. mays. ssp. parviglumis | Mexico |
| TIP485 | TIL10 | Inbred Line | Z. mays. ssp. parviglumis | Mexico |
| TIP508 | TIL12 | Inbred Line | Z. mays. ssp. parviglumis | Mexico |
| TIP517 | TIL15 | Inbred Line | Z. mays. ssp. parviglumis | Mexico |
| TIP534 | TIL11 | Inbred Line | Z. mays. ssp. parviglumis | Mexico |

Further information on these materials is available at www.panzea.org
