# Supplementary material for: From Many, One: Genetic Control of Prolificacy during Maize Domestication
Source: PLoS Genet. 2013 Jun 27;9(6):e1003604. doi: 10.1371/journal.pgen.1003604 (PMC3694832; doi:10.1371/journal.pgen.1003604)
Supplement: Table S5 — Genotypes for the control region in Zea mays ssp. mays, ssp. mexicana and ssp. parviglumis. (DOCX) [file pgen.1003604.s014.docx]

| **subspecies** | **Type** | **Racename/Inbred** | **Country** | **Accession** | **Altitude** | **Latitude** | **Lat Min** | **Longitude** | **Long Min** | ***prol1.1*** | **3’ gt1 indel** |
| --- | --- | --- | --- | --- | --- | --- | --- | --- | --- | --- | --- |
| mays | Landrace Inbred | MR01 (Araguito) | Venezuela | VEN 568 | 183 | 8 | 54 | -64 | 13 | M | M |
| mays | Landrace Inbred | MR02 (Assiniboine) | USA | PI213793 | 457 | 46 | 49 | -100 | 49 | M | T |
| mays | Landrace Inbred | MR03 (Bolita) | Mexico | OAX 68 | 1646 | 17 | 28 | -97 | 18 | M | M |
| mays | Landrace Inbred | MR05 (Cateto) | Bolivia | BOV 635 | 1801 | -16 | 36 | -67 | 15 | M | M |
| mays | Landrace Inbred | MR06 (Chapalote) | Mexico | SIN 2 | 61 | 24 | 48 | -107 | 25 | M | M |
| mays | Landrace Inbred | MR07 (Comiteco) | Mexico | CHS 86 | 1555 | 16 | 15 | -92 | 8 | T | M |
| mays | Landrace Inbred | MR08 (Costeno) | Venezuela | VEN 453 | 146 | 9 | 1 | -63 | 43 | T | M |
| mays | Landrace Inbred | MR09 (Cravo Riogranense) | Brazil | RGS VII | 366 | -30 | 0 | -53 | 0 | T | M |
| mays | Landrace Inbred | MR10 (Cristalino Norteno) | Chile | CHI 349 | 7 | -39 | 55 | -72 | 30 | M | T |
| mays | Landrace Inbred | MR11 (Cuban Flint) | Cuba | CUB 65 | 183 | 23 | 5 | -82 | 40 | T | M |
| mays | Landrace Inbred | MR12 (Havasupai) | USA | PI317675 | 1067 | 36 | 15 | -112 | 40 | M | T |
| mays | Landrace Inbred | MR13 (Hickory King) | USA | PI311237 | • | 37 | 13 | -80 | 25 | M | T |
| mays | Landrace Inbred | MR14 (Longfellow Flint) | Canada | PI217408 | • | 42 | 15 | -71 | 30 | M | T |
| mays | Landrace Inbred | MR17 (Pisankalla) | Bolivia | BOV 344 | 1738 | -21 | 31 | -64 | 45 | M | M/T |
| mays | Landrace Inbred | MR18 (Reventador) | Mexico | NAY 15 | 46 | 22 | 20 | -105 | 23 | M | M |
| mays | Landrace Inbred | MR19 (Santo Domingo) | USA | PI218130 | 1585 | 35 | 31 | -106 | 21 | M | T |
| mays | Landrace Inbred | MR20 (Shoe Peg) | USA | PI269743 | 213 | 36 | 47 | -92 | 13 | M | M |
| mays | Landrace Inbred | MR21 (Tabloncillo) | Mexico | JAL 43 | 1330 | 19 | 59 | -104 | 16 | M | T |
| mays | Landrace Inbred | MR22 (Tuxpeno) | Mexico | TAM 125 | 150 | 22 | 17 | -97 | 53 | T | M |
| mays | Landrace Inbred | MR23 (Zapalote Chico) | Mexico | OAX 70 | 46 | 16 | 20 | -95 | 14 | M | T |
| mays | Landrace Inbred | MR24 (Chullpi) | Peru | AYA 32 | 2350 | -12 | 56 | -74 | 15 | M | M |
| mays | Landrace Inbred | MR25 (Pororo) | Bolivia | BOV 587 | 330 | -16 | 22 | -60 | 57 | M | M |
| mays | Landrace Inbred | MR26 (Pollo) | Colombia | CUN 465 | 1600 | 5 | 3 | -73 | 30 | M | M |
| mays | OP | Arrocillo Amarillo | Mexico | VER 311 | 2220 | 19 | 48 | -97 | 15 | T | M |
| mays | OP | Aysuma | Bolivia | BOV 331 | 2127 | -19 | 2 | -65 | 16 | M | M |
| mays | OP | Canilla | Venezuela | VEN 604 | 183 | 8 | 54 | -64 | 13 | T | M |
| mays | OP | Capia Blanco | Argentina | ARG 499 | • | -23 | 12 | -65 | 21 | M | M |
| mays | OP | Cariaco | Colombia | COR 334 | 200 | 8 | 11 | -76 | 4 | M | M |
| mays | OP | Cateto Nortista Precoce | Surinam | SUR I | 61 | 5 | 45 | -55 | 13 | T | M |
| mays | OP | Cateto Sulino | Uruguay | URG II | 137 | -31 | 42 | -56 | 0 | M | T |
| mays | OP | Chalqueno | Mexico | MEX 48 | 2256 | 19 | 41 | -99 | 8 | M | M |
| mays | OP | Chapalote | Mexico | SIN 2 | 61 | 24 | 48 | -107 | 25 | M | - |
| mays | OP | Chillo | Ecuador | ECU 458 | 2195 | -2 | 51 | -78 | 40 | M | M |
| mays | OP | Chococeno | Ecuador | ECU 964 | 46 | 0 | 40 | -79 | 30 | M | M |
| mays | OP | Conejo | Mexico | GRO 157 | 30 | 17 | 7 | -100 | 28 | T | M |
| mays | OP | Confite Puneno | Peru | PUN 4 | 3900 | -15 | 56 | -69 | 53 | M | M |
| mays | OP | Conico | Mexico | PUE 109 | 2104 | 18 | 14 | -97 | 34 | M | M/T |
| mays | OP | Conico | Mexico | MEX 108 | 2520 | 19 | 6 | -99 | 35 | M | M |
| mays | OP | Coroico | Bolivia | BOV 992 | 200 | -13 | 5 | -64 | 49 | M | M |
| mays | OP | Cuban Flint | Cuba | CUB 63 | 91 | 20 | 40 | -75 | 45 | T | M |
| mays | OP | Dente Paulista | Brazil | SP III | 518 | -22 | 0 | -48 | 0 | T | M |
| mays | OP | Dulce de Jalisco | Mexico | ZAC 182 | 2050 | 22 | 39 | -103 | 42 | M | M/T |
| mays | OP | Dzit Bacal | Mexico | QOO 20 | 10 | 21 | 15 | -86 | 45 | M/T | M |
| mays | OP | Dzit Bacal | Guatemala | GUA 130 | 610 | 14 | 26 | -89 | 40 | M/T | M |
| mays | OP | Guirua | Colombia | MAG 443 | 1860 | 10 | 29 | -73 | 15 | M/T | M |
| mays | OP | Harinoso Tarapaqueno | Chile | CHI 421 | • | -19 | 55 | -69 | 31 | M | M |
| mays | OP | Imbricado | Colombia | CUN 372 | 2625 | 4 | 57 | -74 | 23 | M | M |
| mays | OP | Jala | Mexico | JAL 44 | 1300 | 19 | 57 | -104 | 16 | M | M |
| mays | OP | Karapampa | Bolivia | BOV 961 | 1976 | -21 | 50 | -64 | 8 | M | M |
| mays | OP | Kculli | Peru | CUZ 66 | 3268 | -13 | 32 | -71 | 54 | M | M |
| mays | OP | Montana | Colombia | NAR 426 | 2500 | 1 | 11 | -77 | 15 | M | M |
| mays | OP | Morado | Bolivia | BOV 567 | 2270 | -16 | 28 | -67 | 28 | M | M |
| mays | OP | Motozinteco | Mexico | CHS 650 | 1270 | 15 | 22 | -92 | 15 | T | M/T |
| mays | OP | Mushito | Mexico | MIC 328 | 2200 | 19 | 41 | -102 | 7 | M | M/T |
| mays | OP | Nal-Tel | Mexico | YUC 148 | 30 | 20 | 18 | -89 | 26 | T | M/T |
| mays | OP | Nal-Tel | Mexico | CAM 48 | 50 | 19 | 47 | -90 | 7 | T | M/T |
| mays | OP | Nal-Tel de Altura | Mexico | CHS 196 | 2300 | 15 | 28 | -92 | 9 | T | M |
| mays | OP | Onaveno | Mexico | SON 105 | 820 | 28 | 19 | -108 | 58 | M | M |
| mays | OP | Pepitilla | Mexico | MOR 99 | 1040 | 18 | 41 | -99 | 7 | M/T | M |
| mays | OP | Pira | Venezuela | VEN 485 | 412 | 8 | 0 | -72 | 0 | M | M |
| mays | OP | Pisccorunto | Peru | APC 13 | 2900 | -13 | 40 | -73 | 22 | M | M |
| mays | OP | Sabanero | Colombia | SAN 329 | 2626 | 7 | 19 | -72 | 28 | M | M |
| mays | OP | Serrano | Guatemala | GUA 14 | 1738 | 15 | 17 | -91 | 26 | T | M |
| mays | OP | Serrano Mixe | Mexico | OAX 565 | 2350 | 17 | 8 | -96 | 1 | M/T | M/T |
| mays | OP | Tablilla de Ocho | Mexico | NAY 185 | 1100 | 21 | 41 | -104 | 4 | M | M/T |
| mays | OP | Tuson | Trinidad | TRN 10 | 20 | 10 | 35 | -61 | 7 | T | M |
| mays | OP | Tuxpeno Norteno | Mexico | COA 21 | 1400 | 25 | 26 | -102 | 11 | M | M |
| mays | OP | Tuxpeno Norteno | Mexico | CHH 121 | 1470 | 30 | 54 | -108 | 10 | M/T | M/T |
| mexicana | Inbred | TIL18 | Mexico | CIMMYT 11374 | 2200 | 19 | 21 | -98 | 53 | T | T |
| mexicana | Inbred | TIL25 | Mexico | PUGA 11066 | 1625 | 20 | 22 | -102 | 11 | T | T |
| mexicana | OP | Central Plateau | Mexico | ARROYO ZARCO | 2040 | 19 | 42 | -100 | 34 | T | T |
| mexicana | OP | Central Plateau | Mexico | ARROYO ZARCO | 2040 | 19 | 42 | -100 | 34 | M/T | T |
| mexicana | OP | Central Plateau | Mexico | CIMMYT 5630 | 1920 | 21 | 30 | -101 | 51 | T | T |
| mexicana | OP | Central Plateau | Mexico | CIMMYT 8772 | 1900 | 19 | 55 | -101 | 44 | T | T |
| mexicana | OP | Central Plateau | Mexico | CIMMYT 5630 | 1920 | 21 | 30 | -101 | 51 | T | T |
| mexicana | OP | Central Plateau | Mexico | CIMMYT 8772 | 1900 | 19 | 55 | -101 | 44 | T | T |
| mexicana | OP | Central Plateau | Mexico | CIMMYT 11367 | 1800 | 19 | 54 | -101 | 20 | T | M/T |
| mexicana | OP | Central Plateau | Mexico | CIMMYT 11369 | 1925 | 20 | 10 | -101 | 5 | T | T |
| mexicana | OP | Central Plateau | Mexico | CIMMYT 11370 | 2000 | 20 | 5 | -101 | 31 | T | T |
| mexicana | OP | Central Plateau | Mexico | CIMMYT 11370 | 2000 | 20 | 5 | -101 | 31 | M/T | M/T |
| mexicana | OP | Central Plateau | Mexico | CIMMYT 11371 | 2000 | 20 | 5 | -101 | 31 | T | T |
| mexicana | OP | Central Plateau | Mexico | CIMMYT 11372 | 2100 | 20 | 5 | -101 | 13 | T | T |
| mexicana | OP | Central Plateau | Mexico | CIMMYT 11373 | 2150 | 20 | 3 | -101 | 14 | T | T |
| mexicana | OP | Central Plateau | Mexico | CIMMYT 11367 | 1800 | 19 | 54 | -101 | 20 | T | T |
| mexicana | OP | Central Plateau | Mexico | CIMMYT 11369 | 1925 | 20 | 10 | -101 | 5 | T | T |
| mexicana | OP | Central Plateau | Mexico | CIMMYT 11370 | 2000 | 20 | 5 | -101 | 31 | T | T |
| mexicana | OP | Central Plateau | Mexico | CIMMYT 11371 | 2000 | 20 | 5 | -101 | 31 | T | T |
| mexicana | OP | Central Plateau | Mexico | CIMMYT 11372 | 2100 | 20 | 5 | -101 | 13 | T | T |
| mexicana | OP | Central Plateau | Mexico | CIMMYT 11373 | 2150 | 20 | 3 | -101 | 14 | T | T |
| mexicana | OP | Central Plateau | Mexico | CIMMYT 11394 | 1700 | 20 | 6 | -101 | 54 | T | M |
| mexicana | OP | Central Plateau | Mexico | CIMMYT 11395 | 1810 | 20 | 1 | -101 | 8 | T | T |
| mexicana | OP | Central Plateau | Mexico | CIMMYT 11408 | 2320 | 19 | 24 | -101 | 36 | T | T |
| mexicana | OP | Central Plateau | Mexico | CIMMYT 12825 | 1650 | 19 | 53 | -101 | 13 | M/T | M/T |
| mexicana | OP | Central Plateau | Mexico | CIMMYT 11394 | 1700 | 20 | 6 | -101 | 54 | T | T |
| mexicana | OP | Central Plateau | Mexico | CIMMYT 11394 | 1700 | 20 | 6 | -101 | 54 | M/T | M/T |
| mexicana | OP | Central Plateau | Mexico | CIMMYT 11395 | 1810 | 20 | 1 | -101 | 8 | T | T |
| mexicana | OP | Central Plateau | Mexico | CIMMYT 11408 | 2320 | 19 | 24 | -101 | 36 | T | T |
| mexicana | OP | Central Plateau | Mexico | CIMMYT 12825 | 1650 | 19 | 53 | -101 | 13 | T | T |
| mexicana | OP | Central Plateau | Mexico | CIMMYT 8771 | 1800 | 20 | 9 | -102 | 5 | T | M/T |
| mexicana | OP | Central Plateau | Mexico | CIMMYT 8771 | 1800 | 20 | 9 | -102 | 5 | T | T |
| mexicana | OP | Central Plateau | Mexico | CIMMYT 11066 | 1625 | 20 | 22 | -102 | 11 | T | T |
| mexicana | OP | Central Plateau | Mexico | CIMMYT 11066 | 1625 | 20 | 22 | -102 | 11 | T | T |
| mexicana | OP | Central Plateau | Mexico | JSG Y LOS-3 | 1520 | 20 | 25 | -102 | 21 | T | T |
| mexicana | OP | Central Plateau | Mexico | JSG Y LOS-3 | 1520 | 20 | 25 | -102 | 21 | T | - |
| mexicana | OP | Central Plateau | Mexico | CIMMYT 11392 | 1950 | 24 | 1 | -104 | 29 | T | M |
| mexicana | OP | Central Plateau | Mexico | CIMMYT 11392 | 1950 | 24 | 1 | -104 | 29 | T | M/T |
| mexicana | OP | Central Plateau | Mexico | PI566673 | 1950 | 24 | 11 | -104 | 24 | T | T |
| mexicana | OP | Central Plateau | Mexico | PI566673 | 1950 | 24 | 11 | -104 | 24 | T | T |
| mexicana | OP | Chalco | Mexico | CIMMYT 13788 | 2425 | 19 | 5 | -97 | 32 | M/T | M/T |
| mexicana | OP | Chalco | Mexico | CIMMYT 13788 | 2425 | 19 | 5 | -97 | 32 | T | T |
| mexicana | OP | Chalco | Mexico | CIMMYT 13789 | 2440 | 19 | 4 | -97 | 32 | M/T | M/T |
| mexicana | OP | Chalco | Mexico | CIMMYT 13790 | 2425 | 19 | 8 | -97 | 38 | T | T |
| mexicana | OP | Chalco | Mexico | CIMMYT 13791 | 2355 | 19 | 10 | -97 | 36 | T | M/T |
| mexicana | OP | Chalco | Mexico | CIMMYT 13789 | 2440 | 19 | 4 | -97 | 32 | M/T | T |
| mexicana | OP | Chalco | Mexico | CIMMYT 13790 | 2425 | 19 | 8 | -97 | 38 | M/T | M/T |
| mexicana | OP | Chalco | Mexico | CIMMYT 13791 | 2355 | 19 | 10 | -97 | 36 | T | T |
| mexicana | OP | Chalco | Mexico | CIMMYT 8749 | 2200 | 19 | 13 | -98 | 48 | T | T |
| mexicana | OP | Chalco | Mexico | CIMMYT 8750 | 2340 | 19 | 3 | -98 | 49 | T | T |
| mexicana | OP | Chalco | Mexico | CIMMYT 8751 | 2400 | 19 | 11 | -98 | 53 | M/T | M/T |
| mexicana | OP | Chalco | Mexico | CIMMYT 8752 | 2490 | 19 | 6 | -98 | 53 | T | T |
| mexicana | OP | Chalco | Mexico | CIMMYT 8754 | 2320 | 19 | 1 | -98 | 50 | T | M/T |
| mexicana | OP | Chalco | Mexico | CIMMYT 8768 | 2400 | 19 | 13 | -98 | 59 | T | T |
| mexicana | OP | Chalco | Mexico | CIMMYT 8770 | 2270 | 19 | 19 | -98 | 51 | T | M/T |
| mexicana | OP | Chalco | Mexico | CIMMYT 8749 | 2200 | 19 | 13 | -98 | 48 | T | T |
| mexicana | OP | Chalco | Mexico | CIMMYT 8750 | 2340 | 19 | 3 | -98 | 49 | T | T |
| mexicana | OP | Chalco | Mexico | CIMMYT 8751 | 2400 | 19 | 11 | -98 | 53 | T | T |
| mexicana | OP | Chalco | Mexico | CIMMYT 8752 | 2490 | 19 | 6 | -98 | 53 | T | T |
| mexicana | OP | Chalco | Mexico | CIMMYT 8754 | 2320 | 19 | 1 | -98 | 50 | T | M/T |
| mexicana | OP | Chalco | Mexico | CIMMYT 8768 | 2400 | 19 | 13 | -98 | 59 | T | T |
| mexicana | OP | Chalco | Mexico | CIMMYT 8770 | 2270 | 19 | 19 | -98 | 51 | T | T |
| mexicana | OP | Chalco | Mexico | CIMMYT 11352 | 2200 | 19 | 13 | -98 | 48 | T | M/T |
| mexicana | OP | Chalco | Mexico | CIMMYT 11364 | 2400 | 19 | 12 | -98 | 48 | T | T |
| mexicana | OP | Chalco | Mexico | CIMMYT 11374 | 2200 | 19 | 21 | -98 | 53 | T | T |
| mexicana | OP | Chalco | Mexico | CIMMYT 11352 | 2200 | 19 | 13 | -98 | 48 | T | T |
| mexicana | OP | Chalco | Mexico | CIMMYT 11364 | 2400 | 19 | 12 | -98 | 48 | T | T |
| mexicana | OP | Chalco | Mexico | CIMMYT 11374 | 2200 | 19 | 21 | -98 | 53 | T | T |
| mexicana | OP | Chalco | Mexico | CIMMYT 11386 | 2200 | 19 | 16 | -98 | 55 | T | T |
| mexicana | OP | Chalco | Mexico | CIMMYT 11386 | 2200 | 19 | 16 | -98 | 55 | T | T |
| mexicana | OP | Chalco | Mexico | CIMMYT 11399 | 2390 | 19 | 8 | -98 | 46 | M/T | M/T |
| mexicana | OP | Chalco | Mexico | CIMMYT 11400 | 2200 | 19 | 24 | -98 | 57 | M/T | M/T |
| mexicana | OP | Chalco | Mexico | CIMMYT 11409 | 2200 | 19 | 30 | -98 | 55 | T | T |
| mexicana | OP | Chalco | Mexico | CIMMYT 11409 | 2200 | 19 | 30 | -98 | 55 | T | T |
| mexicana | OP | Chalco | Mexico | CIMMYT 12823 | 2130 | 19 | 15 | -98 | 51 | T | T |
| mexicana | OP | Chalco | Mexico | JFD 643 | 2200 | 19 | 24 | -98 | 57 | T | - |
| mexicana | OP | Chalco | Mexico | CIMMYT 11399 | 2390 | 19 | 8 | -98 | 46 | T | T |
| mexicana | OP | Chalco | Mexico | CIMMYT 11400 | 2200 | 19 | 24 | -98 | 57 | T | M/T |
| mexicana | OP | Chalco | Mexico | CIMMYT 11409 | 2200 | 19 | 30 | -98 | 55 | T | T |
| mexicana | OP | Chalco | Mexico | CIMMYT 12823 | 2130 | 19 | 15 | -98 | 51 | T | T |
| mexicana | OP | Chalco | Mexico | CIMMYT 28620 | 2200 | 19 | 30 | -98 | 55 | T | T |
| mexicana | OP | Chalco | Mexico | CIMMYT 28620 | 2200 | 19 | 30 | -98 | 55 | T | M |
| mexicana | OP | Chalco | Mexico | CIMMYT 8769 | 2300 | 19 | 14 | -99 | 7 | T | T |
| mexicana | OP | Chalco | Mexico | CIMMYT 8769 | 2300 | 19 | 14 | -99 | 7 | T | T |
| mexicana | OP | Chalco | Mexico | CIMMYT 11362 | 2200 | 19 | 15 | -99 | 5 | T | T |
| mexicana | OP | Chalco | Mexico | CIMMYT 11362 | 2200 | 19 | 15 | -99 | 5 | T | T |
| mexicana | OP | Chalco | Mexico | CIMMYT 13792 | 2500 | 19 | 16 | -99 | 27 | M/T | M/T |
| mexicana | OP | Chalco | Mexico | CIMMYT 13793 | 2540 | 19 | 12 | -99 | 33 | T | T |
| mexicana | OP | Chalco | Mexico | CIMMYT 13792 | 2500 | 19 | 16 | -99 | 27 | M/T | M/T |
| mexicana | OP | Chalco | Mexico | CIMMYT 13793 | 2540 | 19 | 12 | -99 | 33 | M/T | M/T |
| mexicana | OP | Nobogame | Mexico | CIMMYT 2438 | 1850 | 26 | 14 | -106 | 58 | T | T |
| mexicana | OP | Nobogame | Mexico | CIMMYT 2438 | 1850 | 26 | 14 | -106 | 58 | T | T |
| mexicana | OP | Nobogame | Mexico | CIMMYT 11387 | 1850 | 26 | 14 | -106 | 58 | T | T |
| mexicana | OP | Nobogame | Mexico | CIMMYT 11387 | 1850 | 26 | 14 | -106 | 58 | T | T |
| mexicana | OP | Nobogame | Mexico | NOBOGAME-2 | 1850 | 26 | 6 | -107 | 0 | M/T | M/T |
| mexicana | OP | Nobogame | Mexico | NOBOGAME-2 | 1850 | 26 | 6 | -107 | 0 | T | T |
| parviglumis | Inbred | TIL01 | Mexico | JSG Y LOS-130 | 1500 | 19 | 36 | -100 | 55 | T | T |
| parviglumis | Inbred | TIL02 | Mexico | JSG Y LOS-119 | 1620 | 18 | 24 | -99 | 58 | T | T |
| parviglumis | Inbred | TIL03 | Mexico | JSG Y MAS-401 | 1405 | 19 | 32 | -104 | 4 | T | T |
| parviglumis | Inbred | TIL04 | Mexico | CIMMYT 8783 | 1580 | 18 | 19 | -99 | 48 | T | T |
| parviglumis | Inbred | TIL05 | Mexico | JSG-197 | 1120 | 16 | 20 | -97 | 2 | T | T |
| parviglumis | Inbred | TIL06 | Mexico | JSG Y LOS-109 | 1260 | 17 | 24 | -99 | 28 | T | T |
| parviglumis | Inbred | TIL07 | Mexico | JSG-378 | 420 | 17 | 11 | -99 | 31 | T | T |
| parviglumis | Inbred | TIL08 | Mexico | JSG-374 | 1580 | 18 | 58 | -99 | 2 | T | T |
| parviglumis | Inbred | TIL09 | Mexico | JSG Y LOS-161 | 1320 | 18 | 54 | -100 | 12 | T | T |
| parviglumis | Inbred | TIL10 | Mexico | CIMMYT 11355 | 1800 | 18 | 24 | -99 | 54 | T | T |
| parviglumis | Inbred | TIL11 | Mexico | JSG Y MAS-264 | 900 | 20 | 48 | -104 | 24 | M | M |
| parviglumis | Inbred | TIL12 | Mexico | PI566686 | 1225 | 18 | 17 | -99 | 11 | T | M |
| parviglumis | Inbred | TIL14 | Mexico | BFB 967 | 1460 | 19 | 33 | -104 | 3 | T | T |
| parviglumis | Inbred | TIL15 | Mexico | TAK SITE 4 | • | 17 | 24 | -99 | 27 | T | T |
| parviglumis | Inbred | TIL16 | Mexico | BK SITE 4 | 1350 | 17 | 25 | -99 | 30 | T | T |
| parviglumis | Inbred | TIL17 | Mexico | W SITE 6 | • | 18 | 21 | -99 | 57 | T | T |
| parviglumis | OP | Balsas | Mexico | JSG-197 | 1120 | 16 | 20 | -97 | 2 | T | T |
| parviglumis | OP | Balsas | Mexico | CIMMYT 11388 | 1120 | 16 | 20 | -97 | 2 | T | T |
| parviglumis | OP | Balsas | Mexico | JSG Y LOS-113 | 1500 | 18 | 19 | -99 | 48 | T | T |
| parviglumis | OP | Balsas | Mexico | PI566688 | 1720 | 18 | 23 | -99 | 58 | T | T |
| parviglumis | OP | Balsas | Mexico | JSG-377 | 625 | 17 | 0 | -99 | 17 | T | T |
| parviglumis | OP | Balsas | Mexico | C-9-78 | 1640 | 18 | 20 | -99 | 48 | T | M |
| parviglumis | OP | Balsas | Mexico | CIMMYT 11401 | 1225 | 18 | 17 | -99 | 11 | T | M/T |
| parviglumis | OP | Balsas | Mexico | IC SITE 3 | 1600 | 18 | 20 | -99 | 50 | T | T |
| parviglumis | OP | Balsas | Mexico | JSG-379 | 765 | 17 | 17 | -99 | 29 | T | T |
| parviglumis | OP | Balsas | Mexico | JSG-378 | 420 | 17 | 11 | -99 | 31 | T | T |
| parviglumis | OP | Balsas | Mexico | BK SITE 4 | 1350 | 17 | 25 | -99 | 30 | T | T |
| parviglumis | OP | Balsas | Mexico | CIMMYT 11353 | 1570 | 18 | 21 | -99 | 47 | T | M/T |
| parviglumis | OP | Balsas | Mexico | CIMMYT 8784 | 1300 | 17 | 24 | -99 | 24 | T | T |
| parviglumis | OP | Balsas | Mexico | JSG Y LOS-109 | 1260 | 17 | 24 | -99 | 28 | T | T |
| parviglumis | OP | Balsas | Mexico | JSG-387 | 1560 | 18 | 21 | -99 | 50 | T | T |
| parviglumis | OP | Balsas | Mexico | CIMMYT 8783 | 1580 | 18 | 19 | -99 | 48 | T | T |
| parviglumis | OP | Balsas | Mexico | JSG-374 | 1580 | 18 | 58 | -99 | 2 | T | T |
| parviglumis | OP | Balsas | Mexico | JSG Y LOS-159 | 1850 | 18 | 57 | -99 | 30 | T | T |
| parviglumis | OP | Balsas | Mexico | JSG-385 | 1795 | 18 | 30 | -99 | 47 | T | T |
| parviglumis | OP | Balsas | Mexico | CIMMYT 11403 | 1720 | 18 | 23 | -99 | 58 | T | T |
| parviglumis | OP | Balsas | Mexico | CIMMYT 8776 | 900 | 17 | 24 | -99 | 28 | T | T |
| parviglumis | OP | Balsas | Mexico | PI566686 | 1225 | 18 | 17 | -99 | 11 | T | M/T |
| parviglumis | OP | Balsas | Mexico | CIMMYT 11355 | 1800 | 18 | 24 | -99 | 54 | T | T |
| parviglumis | OP | Balsas | Mexico | JSG Y LOS-119 | 1620 | 18 | 24 | -99 | 58 | T | T |
| parviglumis | OP | Balsas | Mexico | JSG-382 | 1300 | 17 | 26 | -99 | 29 | M/T | T |
| parviglumis | OP | Balsas | Mexico | CIMMYT 11376 | 1150 | 17 | 17 | -99 | 30 | T | T |
| parviglumis | OP | Balsas | Mexico | PI384064 | 1550 | 18 | 21 | -99 | 51 | T | T |
| parviglumis | OP | Balsas | Mexico | CIMMYT 8758 | 1560 | 18 | 22 | -99 | 46 | T | T |
| parviglumis | OP | Balsas | Mexico | CIMMYT 8779 | 1000 | 19 | 10 | -100 | 19 | T | T |
| parviglumis | OP | Balsas | Mexico | JSG Y LOS-172 | 1620 | 19 | 10 | -100 | 12 | M/T | M/T |
| parviglumis | OP | Balsas | Mexico | JSG Y LOS-178 | 1320 | 19 | 10 | -100 | 19 | T | T |
| parviglumis | OP | Balsas | Mexico | CIMMYT 11402 | 1150 | 18 | 23 | -100 | 4 | T | T |
| parviglumis | OP | Balsas | Mexico | JSG Y LOS-130 | 1500 | 19 | 36 | -100 | 55 | T | T |
| parviglumis | OP | Balsas | Mexico | C-17-78 | 710 | 18 | 50 | -100 | 56 | T | T |
| parviglumis | OP | Balsas | Mexico | C-14-78 | 1200 | 18 | 59 | -100 | 19 | T | T |
| parviglumis | OP | Balsas | Mexico | CIMMYT 8760 | 720 | 18 | 51 | -100 | 54 | T | T |
| parviglumis | OP | Balsas | Mexico | CIMMYT 8763 | 1110 | 19 | 13 | -100 | 17 | M/T | T |
| parviglumis | OP | Balsas | Mexico | CIMMYT 11406 | 880 | 19 | 13 | -100 | 31 | T | T |
| parviglumis | OP | Balsas | Mexico | JSG Y LOS-121 | 1150 | 18 | 23 | -100 | 4 | T | M/T |
| parviglumis | OP | Balsas | Mexico | JSG-193 | 1100 | 19 | 27 | -100 | 54 | T | M/T |
| parviglumis | OP | Balsas | Mexico | CIMMYT 11357 | 740 | 18 | 49 | -100 | 58 | T | M/T |
| parviglumis | OP | Balsas | Mexico | CIMMYT 8762 | 1390 | 18 | 54 | -100 | 9 | T | T |
| parviglumis | OP | Balsas | Mexico | JSG-192 | 1120 | 19 | 22 | -100 | 52 | T | M/T |
| parviglumis | OP | Balsas | Mexico | CIMMYT 11404 | 1180 | 18 | 39 | -100 | 24 | T | M/T |
| parviglumis | OP | Balsas | Mexico | JSG Y LOS-161 | 1320 | 18 | 54 | -100 | 12 | T | T |
| parviglumis | OP | Balsas | Mexico | CIMMYT 11361 | 1235 | 19 | 16 | -100 | 28 | T | T |
| parviglumis | OP | Balsas | Mexico | JSG Y LOS-120 | 1500 | 18 | 22 | -100 | 2 | T | T |
| parviglumis | OP | Balsas | Mexico | JSG-191 | 1000 | 19 | 23 | -100 | 49 | T | M/T |
| parviglumis | OP | Balsas | Mexico | CIMMYT 8767 | 880 | 19 | 13 | -100 | 31 | T | T |
| parviglumis | OP | Balsas | Mexico | JSG Y LOS-176 | 1260 | 19 | 9 | -100 | 23 | T | T |
| parviglumis | OP | Balsas | Mexico | JSG-187 | 740 | 18 | 49 | -100 | 58 | T | T |
| parviglumis | OP | Balsas | Mexico | CIMMYT 8759 | 970 | 18 | 20 | -100 | 7 | T | T |
| parviglumis | OP | Balsas | Mexico | CIMMYT 8782 | 1050 | 18 | 21 | -100 | 11 | T | M/T |
| parviglumis | OP | Balsas | Mexico | JSG-391 | 1420 | 19 | 5 | -100 | 16 | T | T |
| parviglumis | OP | Balsas | Mexico | CIMMYT 8766 | 1040 | 19 | 11 | -100 | 33 | T | M/T |
| parviglumis | OP | Balsas | Mexico | PI566691 | 1100 | 19 | 27 | -100 | 54 | T | T |
| parviglumis | OP | Balsas | Mexico | CIMMYT 8765 | 950 | 19 | 5 | -100 | 28 | T | T |
| parviglumis | OP | Balsas | Mexico | JSG Y LOS-126 | 1100 | 19 | 17 | -100 | 25 | T | T |
| parviglumis | OP | Balsas | Mexico | CIMMYT 8761 | 1120 | 19 | 22 | -100 | 52 | T | M |
| parviglumis | OP | Balsas | Mexico | CIMMYT 11407 | 1500 | 19 | 36 | -100 | 55 | M/T | M/T |
| parviglumis | OP | Jalisco | Mexico | JSG Y LOS-142 | 1460 | 19 | 18 | -103 | 5 | T | T |
| parviglumis | OP | Jalisco | Mexico | JSG Y SMH-355 | 950 | 19 | 57 | -104 | 0 | T | T |
| parviglumis | OP | Jalisco | Mexico | JSG Y MAS-400 | 480 | 19 | 46 | -104 | 45 | T | T |
| parviglumis | OP | Jalisco | Mexico | JSG Y SMH-352 | 1140 | 19 | 55 | -104 | 9 | M/T | M/T |
| parviglumis | OP | Jalisco | Mexico | JSG-203 | 1000 | 19 | 59 | -104 | 3 | M/T | M/T |
| parviglumis | OP | Jalisco | Mexico | BB 967 | 1460 | 19 | 33 | -104 | 3 | T | T |
| parviglumis | OP | Jalisco | Mexico | JSG Y MAS-264 | 900 | 20 | 48 | -104 | 24 | T | M |
| parviglumis | OP | Jalisco | Mexico | JSG Y MAS-402 | 1385 | 20 | 38 | -104 | 25 | T | T |
| parviglumis | OP | Jalisco | Mexico | JSG Y LOS-43 | 1090 | 20 | 48 | -104 | 33 | T | M |
| parviglumis | OP | Jalisco | Mexico | MAS-15 | 1100 | 19 | 27 | -104 | 2 | T | T |
| parviglumis | OP | Jalisco | Mexico | JSG Y LOS-40 | 620 | 20 | 48 | -104 | 35 | T | M/T |
| parviglumis | OP | Jalisco | Mexico | CIMMYT 9477 | 850 | 19 | 49 | -104 | 11 | T | T |
| parviglumis | OP | Jalisco | Mexico | JSG Y LOS-74 | 520 | 19 | 42 | -104 | 49 | T | T |
| parviglumis | OP | Jalisco | Mexico | JSG Y MAS-401 | 1405 | 19 | 32 | -104 | 4 | M/T | M/T |

Further information on these materials is available at [www.panzea.org](http://www.panzea.org)
