# Supplementary material for: From Many, One: Genetic Control of Prolificacy during Maize Domestication
Source: PLoS Genet. 2013 Jun 27;9(6):e1003604. doi: 10.1371/journal.pgen.1003604 (PMC3694832; doi:10.1371/journal.pgen.1003604)
Supplement: Text S1 — Initiation of secondary ear buds. (DOCX) [file pgen.1003604.s015.docx]

To determine if the initiation of secondary ear buds occurs with both the maize and teosinte alleles of *prol1.1*, we dissected primary lateral ear-forming branch buds from the top two nodes of field grown plants with all possible combinations of the 5’ promoter (*prol1.1*) and coding sequence of *gt1* from maize and teosinte (M:M, M:T, T:T and T:M). Serial sections of the entire primary ear-forming branch buds were examined for evidence of secondary bud initiation. Only one primary lateral branch from a genotype containing the 5’ maize *gt1* promoter (M:M or M:T) initiated a rudimentary secondary bud, all others failed to initiate any secondary buds (Table S3). Genotypes containing the teosinte 5’ *gt1* promoter initiated from 2-5 well-developed secondary branches. These data indicate that lines containing the maize allele of *prol1.1* (M:M and M:T) rarely, if at all, initiate secondary ear buds
