# Supplementary material for: From Many, One: Genetic Control of Prolificacy during Maize Domestication
Source: PLoS Genet. 2013 Jun 27;9(6):e1003604. doi: 10.1371/journal.pgen.1003604 (PMC3694832; doi:10.1371/journal.pgen.1003604)
Supplement: Text S2 — Diversity and linkage disequilibrium. (DOCX) [file pgen.1003604.s016.docx]

We used MEGA5 (Tamura et al. 2011) to infer a neighbor-joining (NJ) tree for the region of *prol1.1* (Fig. S5A). Maize and teosinte are clearly separated in the tree, with the exception of 3 individuals: TIL11 (TIP534) was included in the maize cluster, and the maize lines MR11 (BKN20) and MR09 (BKN32) were highly differentiated from other maize. TE insertion polymorphisms are consistent with these groupings, as MR11 (BKN20) and MR09 (BKN32) shared 3 TE insertions with teosinte, TIL11 (TIP534) shared 1 insertions with maize (Fig. S6). Hereafter, we denote the maize- and teosinte-class haplotypes as the class-*M* and class-*T* haplotypes, respectively.

To evaluate patterns of linkage disequilibrium (LD) in maize, we extracted 67 SNPs from the alignment of maize containing 13 class-*M* and 2 class-*T* haplotypes. Fig. 6A in the main text shows the heat map of *r^2^* between all pairs of SNPs. In the middle of the QTL region, we found a block of extremely high LD (called region-*M_LD_* hereafter). We further investigated diversity in the maize region-*M_LD_*. After excluding gaps of in the 13 class-*M* haplotypes, the length of region-*M_LD_* was 2,460 bp. In maize, there are 11 biallelic SNPs in this region (eight of these were not included in the data used for LD in Fig. 6A in the main text because the corresponding bases are missing from the class-*T* haplotype due to TE insertion polymorphism; we also observed one triallelic site which was excluded from further analysis). An NJ tree of region *M_LD_* clearly separates the class*-M* and class*-T* haplotypes, with all class*-M* haplotypes forming a single low-diversity clade (Fig. S5A). Of the 11 SNPs in the maize class-M haplotype, 10 were singletons, resulting in low nucleotide diversity (π = 0.000740) and a Tajima’s *D* of −1.966. We performed 100,000 neutral coalescent simulations under the bottleneck model of Wright et al. (2005) and conditioned on observing 13 and 2 sequences of the two haplotypes. Fewer than 1% of these simulations exhibited diversity statistics as extreme as the observed data, leading us to examine whether a model which included selection might instead better fit the data.

Population genetic analysis of introgression

To rule out the possibility that maize class-*T* or teosinte class-*M* haplotypes were due to recent introgression, we used genotyping data from the maize SNP50 array for these same individuals (Hufford et al. 2012). We first inferred an NJ tree in the software MEGA5 (Takuma et al. 2011) using pairwise genetic distance calculated using all SNPs from chromosome 1 without missing data (Fig. S5B). We also estimated population structure using the software STRUCTURE ver. 2.3.2 (Pritchard et al. 2000), for which we assumed free recombination between all SNPs without missing data, and randomly pruned SNPs closer than 10 kb (other distances were tried with nearly identical results). We ran STRUCTURE under the correlated allele-frequency model with admixture (Falush et al. 2003) for *K* = 2 populations and a burn-in length of 50,000 iterations and a run length of 100,000 iterations (Fig. S5C). Both results show no evidence of recent admixture, with maize and teosinte individuals clearly separated into different clusters.

Haplotype analysis

We estimated the age (*t_M_*) of the class-*M* haploptye using the method of Hudson (2007). We estimate *t_M_* to be ≈13,000 years with a confidence interval of this estimate of 6,000–22,000 years. This estimate overlaps with the time of domestication (9,000 years), and, conditional on our estimates of *s* and *h* (see below), the derived allele arose during the bottleneck in 85% of accepted simulations. The maximum likelihood estimate of *t_M_* = 13, 000 nonetheless appears to be reasonable because both maize and teosinte are polymorphic for both class-*M* and class-*T* haplotypes.

Given the age of the haplotype and an estimated recombination rate, we can approximate the expected length of the haplotype as (*r* × *t_M_* )^−1^, where *r* is the recombination rate per adjacent site per generation. We estimated recombination rate in this region to be 1.1 cM/Mb in the IBM mapping population (Ganal et al. 2011), which provides an expected haplotype length of 7kb. If we instead use the population genetic estimate of the recombination rate *ρ* = 4*Nr*, where *N* is the effective population size, our estimate of π, and assuming ρ/θ = 4.5 in teosinte (θ = 4*Nμ* is population mutation parameters, where *μ* is mutation rate per site per generation; Wright et al. 2005), we obtain an expected length of 1.2 kb. The observed haplotype length of 2.5 kb is thus in line with expectations given the haplotype age.

Simulation and estimation of selection coefficient

We estimated the strength of selection using coalescent simulation. Because we are interested in testing diversity in only one of the two haplotypes present in the maize data, standard coalescent models cannot be applied (Innan and Tajima 1997, 1999). Instead, we simulated a structured coalescent (Nordborg 1997) under a simple bottleneck model of maize domestication (Fig. 6B in the main text). The ancestral population is assumed to be a constant, diploid Wright-Fisher population at equilibrium and of size, *N_A_*. At *t_d_* generations ago, domestication is represented as the beginning of a bottleneck event, which reduces the population size to *N_B_*. At time *t_e_* generations ago the population size recovers to *N_P_*. The duration of bottleneck is specified as *t_B_* = *t_d_* − *t_e_*. We simulated a sample of *n* haploid sequences of length *L* bp. The number of derived and ancestral haplotypes is denoted by *n_d_* and *n_a_*, where *n* = *n_d_* + *n_a_*. Infinite-site mutations and crossing over are incorporated at the rates μ and *r* per site per generation, respectively. The modern frequency of the derived haplotype is denoted by *f*. We assume that the derived allele is favorable after domestication (i.e., after *t_d_*), and denote the selection coefficient and degree of dominance by *s* and *h*, respectively.

For our simulations, we assume *N_A_* = 150,000 (Ross-Ibarra et al. 2009) and μ = 3 × 10^−8^ (Clark et al. 2005). From these two values, expected population mutation parameter is calculated as 4*N_A_*μ = 0.018. This value is consistent with previous estimations of π at silent sites in the teosinte population (e.g., Wright et al. 2005; Ross-Ibarra et al. 2009; Eyre-Walker et al. 1998; Tenaillon et al. 2001, 2004). Archaeological evidence (Piperno 2006) estimates *t_d_* = 9,000 years ago, and we assumed one generation corresponds to one year. The severity of the bottleneck is determined by the ratio *k* = *N_B_*/*t_B_* (Wright et al. 2005; Eyre-Walker et al. 1998; Tenaillon et al. 2004). We use the value *k* = 2.45 estimated by Wright et al. (2005), and follow those authors in assuming *t_B_* = 1,000 generations. The present population size of maize *N_P_* is difficult to estimate, but different values of *N_P_* have little effect on inference of demographic parameters or neutrality tests (Wright et al. 2005; Eyre-Walker et al. 1998; Tenaillon et al. 2004; Gao and Innan 2008; Asano et al. 2011), so we used the simplifying assumption that *N_A_* = *N_P_*. We set *L* = 2,460 bp, and *n_d_* = 13 and *n_a_* = 2 to represent class-*M* and *T* haplotypes in maize, respectively. Finally, we inferred the recombination rate for this region to be 1.1 cM/Mb using a modified genetic map from the IBM mapping population (Ganal et al. 2011) using the cubic spine method implemented in the MayerMap software (Rezvoy et al. 2007).

We performed a structured coalescent simulation using mbs (Teshima and Innan 2009). The initial frequency of the favorable haplotype class (class-*M*) was set to be *f* = 13/15 and its trajectory was simulated backwards in time using binomial sampling with *s* and *h* until fixation of the ancestral allele or until *t_d_* generations ago (solid line of trajectory in Fig. 6B in the main text). If the ancestral allele had not fixed at time *t_d_*, the simulation proceeded with *s* = 0 until fixation of ancestral allele (dashed line of trajectory in Fig. 6B in the main text). Simulations in which the favorable allele fixed backwards in time were discarded. Next, we constructed an ancestral recombination graph and added mutations. The target site of selection was assumed to be in the middle of the simulated region.

We used an approximate likelihood method to estimate the parameters *s* and *h*. The likelihood of the observed summary statistics was estimated as *L*(*s*, *h*) = *P* (Data|*s*, *h*) = *m*’/*m*, where *m* is the number of replicate simulations and *m*’ the number of accepted simulations. We accepted simulations in which *S_ob_* = *S_sim_* ∩ |(*D_ob_* − *D_sim_*)/*D_ob_*| < 0.1, where the subscripts refer to summary statistics within derived (class-*M*) allelic class in observed and simulated data. Simulation proceeded until *m*’ > 10^4^. We also estimated the likelihood for different values of *t_B_* (2,000 and 3,000), *k* (1.8 and 2 from Zhao et al. 2008; Zhao et al. 2011), and *N_P_* (0.5*N_A_* ~ 2*N_A_*). None of these changes meaningfully affected our results. Our simulation suggest reasonably strong selection (*s* = 0.0015) and the degree of dominance (*h* = 1.0) (Fig. 6C in the main text).

Analysis of *gt1* 3’ UTR

Whipple et al. (2011) identified patterns of diversity in the 3’ UTR of the *gt1* transcript consistent with selection. We reanalyzed their data, discovering two clear haplotypes. Similar to *prol1.1*, both haplotypes are present in both maize and teosinte, but differ in frequency (9/16 in the maize population). The class-*M* haplotype, more common in maize, is separated from the class-*T* haplotype by a 41bp deletion at the 5’ end of the UTR. All sequences of the class-*M* haplotype were identical across the entire 604 bp sequences in maize, while the class-*T* haplotype showed normal levels of diversity (S = 19, π = 0.0141). We then applied our coalescent analysis to test whether the frequency and diversity of the class-*M* haplotype could be explained under a neutral demographic model (see above). Coalescent simulations reject the simple neutral model (*P* = 0.0224), suggesting instead selection on the class-*M* haplotype resulting in a partial sweep.

References

1. Tamura, K., Peterson, D., Peterson, N., Stecher, G., Nei, M., et al. (2011) MEGA5: Molecular evolutionary genetics analysis using maximum likelihood, evolutionary distance, and maximum parsimony methods. Mol. Biol. Evol. 28, 2731–2739.

2. Hufford, M. B., Xu, X., van Heerwaarden, J., Pyhäjärvi, T., Chia, J.-M., et al. (2012) Comparative population genomics of maize domestication and improvement. Nat. Gen. 44, 808-811.

3. Pritchard, J. K., Stephens, M. & Donnelly, P. (2000) Inference of population structure using multilocus genotype data. Genetics 155, 945–959.

4. Falush, D., Stephens, M. & Pritchard, J. K. (2003) Inference of population structure using multilocus genotype data: linked loci and correlated allele frequencies. Genetics 164, 1567–1587.

5. Hudson, R. R. (2007) The variance of coalescent time estimates from DNA sequences. J. Mol. Evol. 64, 702–705.

6. Ganal, M. W., Durstewitz, G., Polley, A., Bérard, A., Buckler, E. S., et al. (2011) A large maize (*Zea mays* L.) SNP genotyping array: development and germplasm genotyping, and genetic mapping to compare with the B73 reference genome. PLoS One 6, e28334.

7. Wright, S. I., Bi, I. V., Schroeder, S. G., Yamasaki, M., Doebley, J. F., et al. (2005) The effects of artificial selection on the maize genome. Science 308, 1310–1314.

8. Innan, H. & Tajima, F. (1997) The amounts of nucleotide variation within and between allelic classes and the reconstruction of the common ancestral sequence in a population. Genetics 147, 1431–44.

9. Innan, H. & Tajima, F. (1999) The effect of selection on the amounts of nucleotide variation within and between allelic classes. Genet. Res., Camb. 73, 15–28.

10. Nordborg, M. (1997) Structured coalescent processes on different time scales. Genetics 146, 1501–1514.

11. Ross-Ibarra, J., Tenaillon, M. & Gaut, B. S. (2009) Historical divergence and gene flow in the genus Zea. Genetics 181, 1399–1413.

12. Clark, R. M., Tavaré, S. & Doebley, J. (2005) Estimating a nucleotide substitution rate for maize from polymorphism at a major domestication locus. Mol. Biol. Evol. 22, 2304–2312.

13. Eyre-Walker, A., Gaut, R. L., Hilton, H., Feldman, D. L. & Gaut, B. S. (1998) Investigation of the bottleneck leading to the domestication of maize. Proc. Natl. Acad. Sci. USA 95, 4441–4446.

14. Tenaillon, M. I., Sawkins, M. C., Long, A. D., Gaut, R. L., Doebley, J. F., et al. (2001) Patterns of DNA sequence polymorphism along chromosome 1 of maize (*Zea. mays* ssp. *mays*). Proc. Natl. Acad. Sci. USA 98, 9161–9166.

15. Tenaillon, M. I., U’Ren, J., Tenaillon, O. & Gaut, B. S. (2004) Selection versus demography: a multilocus investigation of the domestication process in maize. Mol. Biol. Evol. 21, 1214–1225.

16. Piperno, D. R. (2006) Quaternary environmental history and agricultural impact on vegetation in Central America. Annals of the Missouri Botanical Garden 93, 274–296.

17. Gao, L. Z. & Innan, H. (2008) Non-independent domestication of the two rice subspecies, *Oryza* *sativa* subsp. *indica* and subsp. *japonica*, demonstrated by multilocus microsatellites. Genetics 179, 965–976.

18. Asano, K., Yamasaki, M., Takuno, S., Miura, K., Katagiri, S., et al. (2011) Artificial selection for a green revolution gene during japonica rice domestication. Proc. Natl. Acad. Sci. USA 108, 11034–11039.

19. Rezvoy, C., Charif, D., Guéguen, L. & Marais, G. A. (2007) MareyMap: an R-based tool with graphical interface for estimating recombination rates. Bioinformatics 23, 2188–2189.

20. Teshima KM, Innan H. BMC Bioinformatics. (2009) mbs: modifying Hudson's ms software to generate samples of DNA sequences with a biallelic site under selection.10:166.

21. Zhao, Q., Thuillet, A. C., Uhlmann, N. K., Weber, A., Rafalski, J. A., et al. (2008) The role of regulatory genes during maize domestication: evidence from nucleotide polymorphism and gene expression. Genetics 178, 2133–2143.

22. Zhao, Q., Weber, A. L., McMullen, M. D., Guill, K. & Doebley, J. (2011) MADS-box genes of maize: frequent targets of selection during domestication. Genet. Res. (Camb) 93, 65–75.
